# Kinesio Taping for Managing Postoperative Sequelae of Mandibular Third Molar Impaction: A Prospective Clinical Study

**DOI:** 10.7759/cureus.93716

**Published:** 2025-10-02

**Authors:** Upika Jain, Durga Shanker Gupta, Arghya Upadhaya, Kumari Aparajita, Subham Mukhopadhyay, Pragya Sharma, Seema Gupta

**Affiliations:** 1 Department of Oral and Maxillofacial Surgery, Teerthanker Mahaveer Dental College and Research Centre, Moradabad, IND; 2 Department of Orthodontics, Kothiwal Dental College and Research Centre, Moradabad, IND

**Keywords:** impacted, kinesiology, pain, quality of life, third molar, trismus

## Abstract

Introduction: Lower third molar surgery is a common oral procedure often accompanied by postoperative complications, such as pain, swelling, trismus, and reduced quality of life (QoL). Despite the standard care with analgesics and antibiotics, these issues can persist, prompting the exploration of adjunctive therapies. Kinesio taping (KT; Kinesio Holding Corporation, Albuquerque, NM), widely used for over 15 years to alleviate pain and inflammation, offers a non-invasive option. This study investigated the efficacy of KT in enhancing recovery after mandibular third molar extraction.

Materials and methods: This prospective clinical study was conducted over 18 months at the Department of Oral and Maxillofacial Surgery, Teerthanker Mahaveer Dental College and Research Centre, Moradabad, India. A total of 36 patients aged 18-40 years with impacted mandibular third molars (Pederson Difficulty Index = 3-7; American Society of Anesthesiologists grade 1) were allocated to two groups (n = 18 each) using a lottery method. Group 1 received analgesics and antibiotics only, while group 2 received analgesics, antibiotics, and KT applied extra-orally. KT was performed by a certified therapist from the clavicle to the infraorbital rim, with 20% tension, and was left in place for five days. Outcomes, including pain assessed via the Visual Analog Scale (VAS) scores, swelling assessed via five-point measurements, trismus assessed via interincisal distance, and QoL assessed via short-form Oral Health Impact Profile-14 (OHIP-14), were evaluated preoperatively and on days one, three, and seven. Statistical analysis was performed using the Mann-Whitney U test, Friedman test, independent t-test, and repeated-measures analysis of variance (ANOVA) (p < 0.05).

Results: The mean ages of group 1 (29.94 ± 6.38 years) and group 2 (27.72 ± 5.37 years) were comparable (p = 0.266). Pain was significantly lower in group 2 on day seven (2.22 ± 0.73 vs. 1.67 ± 0.77, p = 0.047). Swelling was reduced in group 2 on day one (13.71 mm vs. 12.65 mm, p = 0.001), but not on days three or seven (p > 0.05). Trismus showed no significant intergroup difference (p > 0.05), although intragroup improvement was observed (p < 0.05). QoL scores were significantly better in group 2 on days one (16.45 ± 3.45 vs. 9.56 ± 3.15, p = 0.001), three (11.25 ± 2.35 vs. 5.65 ± 2.34, p = 0.001), and seven (8.45 ± 2.15 vs. 3.45 ± 2.12, p = 0.001).

Conclusion: KT effectively reduced early postoperative pain and swelling, and improved QoL following lower third molar surgery, although its impact on improvement in mouth opening was limited. These findings support KT as a valuable adjunct, warranting further large-scale blinded studies to optimize its application.

## Introduction

Lower third molar surgery, commonly referred to as wisdom tooth extraction, is one of the most frequently performed oral surgeries worldwide. Despite its commonality, the procedure is associated with notable postoperative complications, including pain, swelling, trismus (restricted jaw movement), and subsequent impact on patients’ quality of life (QoL) [1]. These complications can prolong recovery, disrupt daily activities, and lead to discomfort, which affects both physical and psychological well-being. Conventional postoperative management often includes analgesics, anti-inflammatory medications, and cold compresses; however, these approaches may not fully address all symptoms, particularly trismus and prolonged swelling [2]. As a result, there is growing interest in adjunctive therapies to enhance recovery outcomes.

Kinesiology tape, such as Kinesio taping (KT; Kinesio Holding Corporation, Albuquerque, NM), an elastic adhesive tape designed to support muscles, reduce inflammation, and improve circulation, has emerged as a promising intervention [3]. Originally developed for sports injuries, KT is increasingly being applied in post-surgical settings because of its ability to facilitate lymphatic drainage and reduce tissue pressure [3,4]. By lifting the skin, the tape promotes blood flow and reduces edema, potentially alleviating pain and swelling. Furthermore, its supportive properties may enhance jaw mobility and address trismus, which is a significant concern following third molar surgery [5]. A systematic review by Wang et al. [6] highlighted its potential in pain management, whereas Firoozi et al. [7] noted that KT reduces postoperative pain and discomfort after third molar extractions until the seventh postoperative day. Similarly, Qi et al. [8] indicated that KT may alleviate pain and has demonstrated beneficial effects; however, there is a lack of compelling evidence to support its efficacy in diminishing edema following surgical removal of the third molar. Therefore, the overall efficacy of KT in the context of lower-third molar surgery remains underexplored, with limited comprehensive studies evaluating its impact across multiple parameters.

This study sought to address this gap by systematically evaluating the efficacy of KT in the postoperative period following lower third molar surgery. By focusing on key parameters, such as pain, swelling, trismus, and QoL, this study aimed to provide evidence-based insights into the potential benefits of KT as an adjunctive therapy. The objective of this study was to assess the efficacy of KT in reducing postoperative pain, swelling, and trismus, as well as its influence on improving patients’ QoL, thereby contributing to optimized recovery protocols for patients undergoing oral surgery.

## Materials and methods

This prospective clinical study was conducted at the Outpatient Department of Oral and Maxillofacial Surgery, Teerthanker Mahaveer Dental College and Research Centre, Moradabad, India, over a period of 18 months from August 2023 to January 2024. Ethical approval was obtained from the Institutional Ethics Committee (Reference: TMDCRC/IEC/TH/22-23/OMFS 04), ensuring compliance with the ethical principles outlined in the Declaration of Helsinki (1975, revised 2013). All patients provided written informed consent prior to enrolment. This study does not constitute a clinical trial requiring registration, as it employs KT, a well-established intervention used for over 15 years to reduce pain and inflammation in various clinical settings, including postsurgical recovery, and does not investigate a novel application.

Patients aged 18 to 40 years with partially or completely impacted mandibular third molars, as indicated by the Pederson Difficulty Index (score 3-7) [9], were included, provided that they were systemically healthy (American Society of Anesthesiologists grade 1) and willing to participate [10]. Exclusion criteria included acute symptoms (pain, swelling, trismus, or fever), compromised health conditions (such as cardiac, hepatic, renal disorders, or autoimmune diseases), pregnancy, lactation, or allergies to KT.

A priori power analysis was conducted using the G*Power software (v.3.6.9, Heinrich Heine University Düsseldorf, Düsseldorf, Germany). To detect an effect size of 0.8 (as reported in a prior study comparing Visual Analog Scale (VAS) scores) with 80% power and a 5% alpha level for an independent t-test, a total sample size of 36 patients (18 per group) was determined to be necessary [5].

A total of 36 patients were assigned to two equal groups (18 each) using a lottery method with even and odd numbers. Group 1 received analgesics and antibiotics only, while group 2 received analgesics, antibiotics, and KT (Kinesio Holding Corporation, Albuquerque, NM) applied extra-orally post surgery.

**Figure 1:**
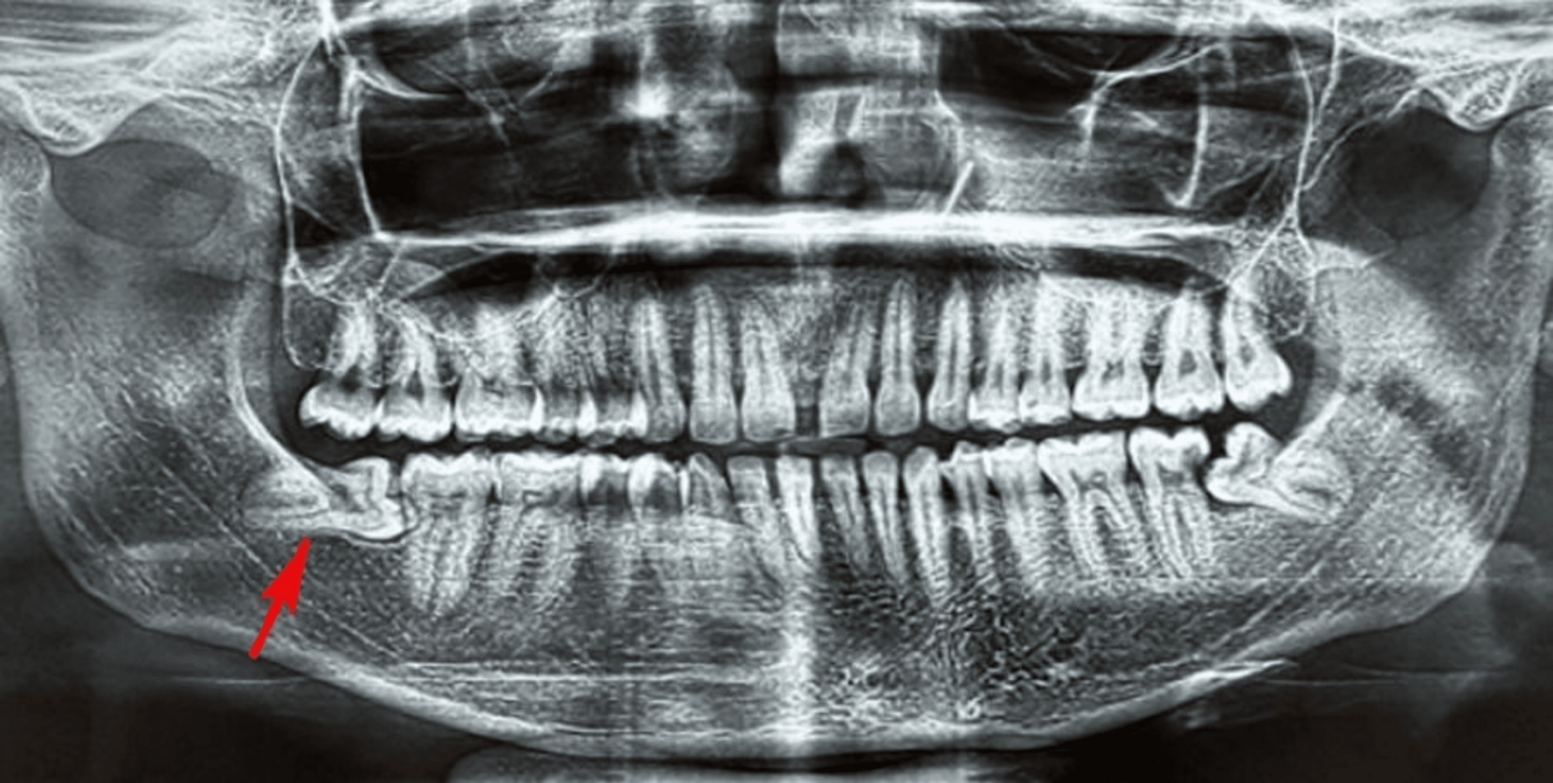
Orthopantomogram showing an impacted mandibular third molar tooth on the right side (red arrow).

Surgical procedures were standardized for both groups. Detailed clinical history and radiological evaluation using panoramic radiography or cone-beam computed tomography (CBCT) were performed preoperatively (Figure 1). Patients were prepared under aseptic conditions with povidone-iodine (5% w/v; Betadine®, Win-Medicare Pvt. Ltd., New Delhi, India). Local anesthesia was administered using a 2 mL syringe with 2% lidocaine and 1:80,000 adrenaline (Lignox®, Indoco Remedies Ltd., Mumbai, India) via an inferior alveolar nerve block and long buccal nerve block. A Ward’s or modified Ward’s incision was made, followed by bone guttering with a round bur and a straight fissure bur under continuous saline irrigation. Tooth sectioning was performed as needed, and the socket was irrigated with normal saline (0.9%; Baxter India Pvt. Ltd., Gurugram, India). Primary closure was achieved using 3-0 black silk sutures (Ethicon®; Johnson & Johnson, Mumbai, India).

Postoperatively, group 2 received KT stretching from above the clavicle to the area of greatest swelling, whereas group 1 did not. All taping procedures were conducted by a single investigator, who was a certified K-taping therapist. Prior to application, the skin was thoroughly cleaned to remove moisture and oil, and shavings were performed if necessary to ensure proper adhesion. The tape used was skin-colored and measured 50 mm × 5 mm. The tape length was individually tailored for each patient and measured from the clavicle to the point of maximum swelling on the face. The tape ends were rounded off to prevent lifting. The adhesive backing was carefully removed to minimize contact with the medical-grade acrylic adhesive, and the base was positioned just above the supraclavicular lymph node area for direct lymphatic drainage. The patients were placed in a stretched position during the application.

The tape strip was applied with a slight tension of approximately 20%, extending across the cervical, submental, mandibular, submandibular, preauricular, and parotid lymph node regions, crossing the zygomatic arch, and reaching toward the infraorbital rim. Following the application, the tape was gently rubbed to activate the adhesive. The tape remained in place for at least five days, with edges trimmed if any lifting occurred prior to removal.

Both groups were prescribed amoxicillin 500 mg + clavulanic acid 125 mg (Augmentin®, GlaxoSmithKline Pharmaceuticals Ltd., Mumbai, India) every 12 hour, metronidazole 400 mg (Flagyl®, Abbott India Ltd., Mumbai, India) every eight hour, acetaminophen 500 mg (Dolo®, Micro Labs Ltd., Bengaluru, India) every eight hour, and pantoprazole 40 mg (Pantocid®, Sun Pharmaceutical Industries Ltd., Mumbai, India) daily for five days. Sutures were removed on the seventh postoperative day.

**Figure 2:**
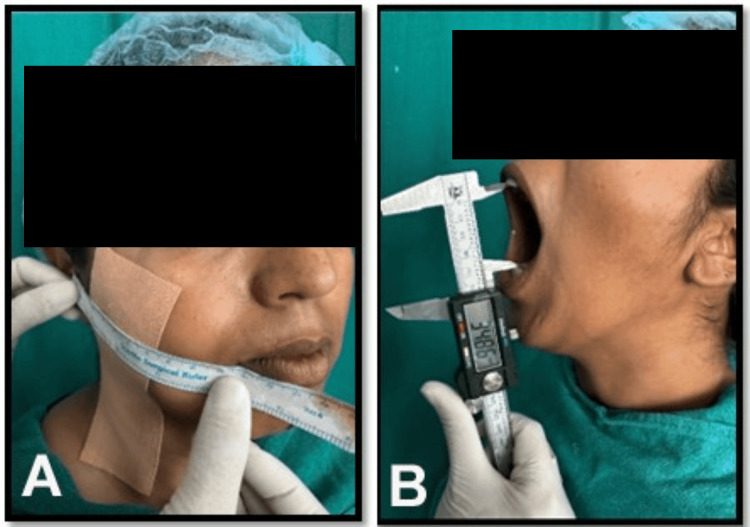
(A) Measurement of facial swelling. (B) Measurement of mouth opening with a vernier caliper.

Outcome assessments were conducted on the first, third, and seventh postoperative days. Pain was evaluated using VAS scores, as given by Downie et al. [11], where patients rated discomfort from 0 (no pain) to 10 (severe pain). Facial swelling was measured as described by UStün et al. [12], using a surgical scale and marker, calculating the mean of five linear measurements between seven reference points: tragus to corner of lip, tragus to soft tissue pogonion, tragus to eye lateral canthus, eye lateral canthus to inferior point on the angle of the lower jaw, and inferior point on the angle of the lower jaw to the midpoint of the nasal bone (Figure 2A). Changes were compared to preoperative baseline values to assess swelling regression. Trismus was also assessed as mentioned by UStün et al. [12], as maximum mouth opening, measured as the interincisal distance between the right central incisors with a metallic scale or vernier caliper, comparing postoperative values (normal range: 35-55 mm) to preoperative baselines (Figure 2B). The short-form Oral Health Impact Profile-14 (OHIP-14) by Slade [13] was used to assess physical, psychological, and social well-being (quality of life). Formal written permission, including licensing, was obtained from the respective publishers for the use of the Pederson's Difficulty Index, the facial swelling measurement scheme, and the short-form OHIP-14.

OHIP-14 is a validated questionnaire consisting of 14 items across seven domains: functional limitation, physical pain, psychological discomfort, physical disability, psychological disability, social disability, and handicap. Each item assesses the frequency of negative impacts on QoL, with responses scored on a five-point Likert scale ranging from 0 ("never") to 4 ("very often"). The total OHIP-14 score is calculated by summing the responses to all 14 items, yielding a range of 0 to 56. Higher scores indicate greater frequency and severity of adverse impacts, reflecting poorer QoL, while lower scores signify better QoL with fewer disruptions to physical, psychological, and social well-being. To ensure reliability, all measurements were performed by two trained examiners, with inter-examiner calibration conducted prior to the study using duplicate measurements in 10 patients, achieving an intraclass correlation coefficient of ≥0.85. Data were recorded systematically.

Statistical analyses were performed using SPSS software (version 20, IBM Corp., Armonk, NY). The normality of continuous data was assessed. As the pain data were non-normally distributed, the Mann-Whitney U test was used for intergroup comparisons, and the Friedman test was used for comparisons across multiple time points. For swelling, trismus, and QoL, which were normally distributed, intergroup comparisons were made using an independent t-test, while intragroup comparisons across time were analyzed using repeated-measures analysis of variance (ANOVA). For all tests, a post-hoc Bonferroni analysis was applied, and a p-value of < 0.05 was considered statistically

## Results

Analysis showed that the mean age in group 1 was 29.94 ± 6.38 years, compared to 27.72 ± 5.37 years in group 2, with no statistically significant difference (p = 0.266). Furthermore, the distribution of sex was similar between the groups, with 10 (55.6%) males in group 1 versus seven (38.9%) in group 2 (p = 0.317). These results demonstrated that the two groups were well matched in terms of age and sex (Table 1).

**Table 1:** Demographic details of the study samples. Group 1: Patients receiving analgesics only. Group 2: Patients receiving analgesics with Kinesio taping (KT). P > 0.05 denotes no statistical significance. Test statistics chi: The chi-square test of independence is used for sex distribution. Test statistics t: An independent t-test is used for comparison of age. Age is presented as mean ± standard deviation (SD), whereas sex distribution is presented as frequency (n) and percentage (%), where n denotes the number of patients in each group.

Parameters		Group 1	Group 2	Test statistics	p-value
Age	Years (mean ± SD)	29.94 ± 6.38	27.72 ± 5.37	t = 1.13	0.266
Sex	Male, n (%)	10 (55.6)	7 (38.9)	chi = 1.01	0.317
	Female, n (%)	8 (44.4)	11 (61.1)		

Although no significant intergroup differences were observed preoperatively (p = 0.064), on day one (p = 0.171), or on day three (p = 0.118), a statistically significant difference was identified on day seven. At this interval, group 1 reported a statistically significantly higher mean pain VAS score (2.22 ± 0.73) than group 2 (1.67 ± 0.77). This finding suggests that the intervention or characteristic defining group 2 was associated with significantly lower reported pain levels by the end of the first postoperative week (Table 2).

**Table 2:** Comparison of the Visual Analog Scale (VAS) pain scores between groups at multiple time intervals using the Mann-Whitney U test. Group 1: Patients receiving analgesics only. Group 2: Patients receiving analgesics with Kinesio taping (KT). * P < 0.05 denotes statistical significance using the Mann-Whitney U test. Pain was assessed using the Visual Analog Scale (VAS) by Downie et al. [11], ranging from 0 (no pain) to 10 (severe pain), a tool freely available for research use. VAS scores were reported as mean, median, standard deviation (SD), and mean rank for non-parametric analyses.

Time interval	Groups	n	Mean	Median	SD	Mean rank	U statistics	p-value
Preoperative pain scores	Group 1	18	2.61	3	0.92	21.75	103.5	0.064
	Group 2	18	2.60	2	0.91	15.25		
Pain scores on postoperative day 1	Group 1	18	2.78	3	0.65	20.94	118.0	0.171
	Group 2	18	2.44	2	0.51	16.06		
Pain scores on postoperative day 3	Group 1	18	2.83	3	0.71	21.25	112.5	0.118
	Group 2	18	2.44	2	0.62	15.75		
Pain scores on postoperative day 7	Group 1	18	2.22	2	0.73	22.00	99.0	0.047*
	Group 2	18	1.67	1.5	0.77	15.00		

Preoperatively, the mean swelling was comparable between group 1 (11.87 mm) and group 2 (11.93 mm), with no significant difference (p = 0.817), confirming baseline equivalence. A highly significant difference emerged on day one; group 1 exhibited a markedly higher mean swelling (13.71 mm) than group 2 (12.65 mm), with a mean difference of 1.05 mm (p = 0.001). This significant disparity was not maintained at subsequent time points, as comparisons on days three (p = 0.09) and seven (p = 0.107) showed no statistically significant differences between the groups. This pattern suggests that the intervention or characteristic defining group 2 was effective in significantly reducing swelling in the immediate postoperative period (day one); however, its effect on this specific outcome was not sustained throughout the first week (Table 3).

**Table 3:** Comparison of the swelling size (mm) between groups at multiple time intervals using an independent t-test. Group 1: Patients receiving analgesics only. Group 2: Patients receiving analgesics with Kinesio taping (KT). * P < 0.05 denotes statistical significance using an independent t-test. Facial swelling was measured in millimeters (mm) using the protocol described by UStün et al. [12], with results reported as mean and standard deviation (SD). Permission to use this measurement method was obtained from Elsevier. The mean difference was calculated by subtracting group 2’s measurements from group 1’s measurements.

Time intervals	Groups	n	Mean	SD	Mean difference (mm)	t value	p-value
Preoperative	Group 1	18 (100%)	11.87	0.91	-0.06	-0.23	0.817
	Group 2	18 (100%)	11.93	0.70			
Postoperative day 1	Group 1	18 (100%)	13.71	0.83	1.05	4.40	0.014*
	Group 2	18 (100%)	12.65	0.59			
Postoperative day 3	Group 1	18 (100%)	13.62	0.75	0.67	1.75	0.091
	Group 2	18 (100%)	12.95	1.44			
Postoperative day 7	Group 1	18 (100%)	12.56	1.06	0.64	1.65	0.107
	Group 2	18 (100%)	11.92	1.25			

Preoperatively, the mean mouth opening measurements were comparable between group 1 (37.91 mm) and group 2 (39.12 mm), with no significant difference (p = 0.545), establishing baseline equivalence. No statistically significant intergroup differences were found at any subsequent postoperative time point: day one (p = 0.335), day three (p = 0.112), and day seven (p = 0.172). Although group 2 consistently demonstrated a numerically higher mean mouth opening at every assessment, these differences did not reach statistical significance (Table 4).

**Table 4:** Comparison of trismus as maximum mouth opening (mm) between groups at multiple time intervals using an independent t-test. Group 1: Patients receiving analgesics only. Group 2: Patients receiving analgesics with Kinesio taping (KT). P > 0.05 denotes no statistical significance using an independent t-test. Trismus is measured as maximum interincisal distance (mouth opening), described by UStün et al. [12], in millimeters, and presented as mean and standard deviation (SD). The mean difference is calculated by subtracting the reading of group 2 from group 1.

Time intervals	Groups	n (100%)	Mean	SD	Mean difference (mm)	t value	p-value
Preoperative	Group 1	18 (100%)	37.91	5.89	-1.21	-0.61	0.545
	Group 2	18 (100%)	39.12	5.98			
Postoperative day 1	Group 1	18 (100%)	33.13	6.44	-2.04	-0.98	0.335
	Group 2	18 (100%)	35.18	6.09			
Postoperative day 3	Group 1	18 (100%)	32.78	5.95	-3.11	-1.63	0.112
	Group 2	18 (100%)	35.89	5.47			
Postoperative day 7	Group 1	18 (100%)	36.36	4.74	-2.22	-1.40	0.172
	Group 2	18 (100%)	38.58	4.81			

Intragroup analysis across time intervals revealed significant changes in all outcome measures in both groups. For pain, the Friedman test showed a significant effect of time in both groups. Similarly, a repeated-measures ANOVA identified statistically significant temporal variations in swelling and trismus in both groups. This implies that irrespective of the group assignment, all three parameters (pain, swelling, and trismus) underwent significant evolution throughout the postoperative period (Table 5).

**Table 5:** Comparison of outcome measures between multiple time intervals within study groups. Group 1: Patients receiving analgesics only. Group 2: Patients receiving analgesics with Kinesio taping (KT). * P < 0.05 denotes statistical significance using repeated-measures analysis of variance (ANOVA) for trismus and swelling, and the Friedman test for pain scores. Pain was assessed using the Visual Analog Scale (VAS) score, according to Downie et al. [11]. Facial swelling and trismus measurements were assessed according to UStün et al. [12].

Groups	Time intervals	Pain (VAS scores)		Swelling (mm)		Trismus as assessed from maximum mouth opening (mm)	
		Friedman chi-square stats	p-value	F-value	p-value	F-value	p-value
Group 1	Preoperative	9.95	0.019*	36.63	0.001*	19.69	0.012*
	Postoperative day 1						
	Postoperative day 3						
	Postoperative day 7						
Group 2	Preoperative	14.75	0.002*	6.75	0.001*	15.27	0.001*
	Postoperative day 1						
	Postoperative day 3						
	Postoperative day 7						

The post-hoc Bonferroni analysis revealed specific temporal changes within each group. For pain, a significant reduction occurred between days one and seven (group 1: p = 0.047; group 2: p = 0.011) and days three and seven (group 1: p = 0.018; group 2: p = 0.004) in both groups. Swelling increased significantly from preoperative levels to day one and day three in both groups (all p < 0.05), but subsequently decreased significantly from day one to day seven only in group 1 (p = 0.001). For trismus, mouth opening significantly improved from preoperative values to all postoperative days in group 1 (all p < 0.05) and on days one and three in group 2. It then improved significantly from day one to day seven in both groups (group 1, p = 0.017; group 2, p = 0.004), as shown in Table 6.

**Table 6:** Pairwise comparison for outcome measures at different time intervals with the Bonferroni post-hoc test. Group 1: Patients receiving analgesics only. Group 2: Patients receiving analgesics with Kinesio taping (KT). * P < 0.05 denotes statistical significance using post-hoc analysis with the Bonferroni test. Pain was assessed using the Visual Analog Scale (VAS) score, according to Downie et al. [11]. Facial swelling and trismus measurements were assessed according to UStün et al. [12].

Pairwise groups		Group 1		Group 2	
		Test statistics	p-value	Test statistics	p-value
Preoperative pain	Pain on day 1	-0.50	0.981	-0.81	0.245
Preoperative pain	Pain on day 3	-0.64	0.551	-0.92	0.133
Preoperative pain	Pain on day 7	0.58	0.701	0.50	0.981
Pain on day 1	Pain on day 3	-0.14	0.999	-0.11	0.999
Pain on day 1	Pain on day 7	1.08	0.047*	1.31	0.011*
Pain on day 3	Pain on day 7	1.22	0.018*	1.42	0.004*
Preoperative swelling	Swelling on day 1	-8.96	0.001*	-5.70	0.001*
Preoperative swelling	Swelling on day 3	-9.21	0.001*	-3.23	0.031*
Preoperative swelling	Swelling on day 7	-2.55	0.124	0.05	0.999
Swelling on day 1	Swelling on day 3	0.61	0.999	-1.07	0.999
Swelling on day 1	Swelling on day 7	5.25	0.001*	2.53	0.131
Swelling on day 3	Swelling on day 7	5.62	0.001*	2.88	0.062
Preoperative mouth opening	Mouth opening on day 1	5.25	0.001*	5.74	0.001*
Preoperative mouth opening	Mouth opening on day 3	5.42	0.001*	3.75	0.011*
Preoperative mouth opening	Mouth opening on day 7	3.25	0.028*	0.85	0.999
Mouth opening on day 1	Mouth opening on day 3	0.86	0.999	-1.52	0.877
Mouth opening on day 1	Mouth opening on day 7	-3.49	0.017*	-4.17	0.004*
Mouth opening on day 3	Mouth opening on day 7	-3.93	0.006*	-3.89	0.007*

A comparison of QoL scores between the groups is presented in Table 7. A statistically significant difference was observed at each postoperative time point. On day one, group 1 reported a significantly higher mean score (16.45 ± 3.45) than group 2 (9.56 ± 3.15; p = 0.001). This significant disparity persisted on day three (11.25 ± 2.35 vs. 5.65 ± 2.34; p = 0.001) and day seven (8.45 ± 2.15 vs. 3.45 ± 2.12; p = 0.001). The inference is that patients in group 2 consistently experienced superior QoL throughout the first postoperative week, as indicated by their significantly lower scores on the measured scale.

**Table 7:** Comparison of quality-of-life (QoL) scores using the short-form Oral Health Impact Profile-14 (OHIP-14) questionnaire between groups at first, third, and seventh postoperative days. Group 1: Patients receiving analgesics only. Group 2: Patients receiving analgesics with Kinesio taping (KT). * P < 0.05 denotes statistical significance using an independent t-test. QoL scores are presented as mean ± standard deviation (SD), using the OHIP-14 questionnaire by Slade [13], with permission for use obtained from John Wiley & Sons.

Time intervals	Group 1	Group 2	T statistics	p-value
	Mean ± SD	Mean ± SD		
Postoperative day 1	16.45 ± 3.45	9.56 ± 3.15	12.34	0.015*
Postoperative day 3	11.25 ± 2.35	5.65 ± 2.34	10.45	0.001*
Postoperative day 7	8.45 ± 2.15	3.45 ± 2.12	14.32	0.018*

## Discussion

The findings of this study provided valuable insights into the efficacy of KT as an adjunctive therapy following lower third molar surgery, particularly in reducing pain and swelling, and improving QoL, although its impact on trismus was less pronounced. The significant reduction in pain scores in group 2 (KT group) by day seven (p = 0.047) aligns with previous research demonstrating KT’s analgesic effects [5-8].

KT was performed after stretching. The tape reverts to its initial length after its application to the epidermis, producing a tensile force on the dermal surface that induces skin convolutions. This phenomenon results in augmentation of the interstitial spacing between the epidermis and the underlying connective tissue, thereby enhancing the circulation of both blood and lymphatic fluids. Consequently, this alleviates the pressure exerted on nociceptive receptors at the location of KT application, leading to a reduction in pain perception [6]. Jaron et al. [14] conducted a systematic review to assess the effects of KT on postoperative complications after third molar extraction and reported that KT is effective in reducing postoperative pain and edema.

KT enhances pain relief by facilitating lymphatic drainage and reducing tissue pressure, a mechanism supported by the current study’s observation of lower VAS scores in the KT group over time [6]. The initial pain reduction from day one to day seven in both groups (p < 0.05) is consistent with the natural resolution of postoperative inflammation; however, the sustained lower pain in group B suggests an additive benefit of KT, possibly due to its prolonged application over five days. In the present investigation, KT was applied for a duration of five consecutive days, in accordance with a previous study [15], which indicated that the tensile efficacy of KT ranges from three to five days.

The swelling results further corroborate the efficacy of KT, with a significant reduction observed on day one in group 2 (p = 0.001) compared to that in group 1. This finding was in accordance with a study by Patil et al. [5], who noted KT’s ability to minimize edema by improving lymphatic flow, particularly in the immediate postoperative phase. The lack of sustained significance on days three and seven (p > 0.05) may reflect the natural regression of swelling over time, as both groups showed significant decreases by day seven, suggesting that KT’s effect is most pronounced early in recovery. The tailored application from the clavicle to the infraorbital rim likely optimized lymphatic drainage, supporting the observed initial benefit.

The lack of significant intergroup differences in trismus contrasts with previous studies, which reported a significant improvement in mouth opening with KT [5-8,16]. This discrepancy may stem from the study’s focus on interincisal distance rather than functional jaw movement or the uniform surgical technique limiting trismus variability. Intragroup improvements from day one to day seven (p < 0.05) indicated a general recovery trend, but KT’s limited impact suggests that it may not sufficiently address the mechanical restrictions post surgery.

QoL scores revealed a significant advantage for group 2 across all postoperative days (p = 0.001), aligning with Jaron et al. [17], who linked reduced postoperative symptoms to enhanced well-being. The lower OHIP-14 scores in group 2 reflect reduced physical and psychological burdens, likely driven by lower pain and swelling, underscoring KT’s holistic benefit. The use of the validated OHIP-14 [13] ensured a robust QoL assessment.

Clinically, these findings suggest that KT can be integrated into postoperative care protocols to accelerate recovery and improve patient satisfaction, particularly for pain and swelling management. Its non-invasive nature and established safety profile make it a practical adjunct. However, limitations include the non-blinded design, which may introduce bias, and the small sample size, which potentially limits generalizability. KT is an external, visible intervention, and it is virtually impossible to blind the operator and the patient, introducing a high risk of performance and detection bias. Future studies are needed that employ more robust, randomized, blinded designs (e.g., using a sham/placebo tape application) to fully validate the findings. The lack of long-term follow-up beyond day seven also restricts insights into sustained effects.

## Conclusions

This study demonstrated that KT served as an effective adjunctive therapy in the postoperative management of lower third molar surgery, particularly in reducing pain and swelling and enhancing QoL during the initial recovery phase. The significant reduction in pain scores on day seven and swelling on day one in the KT group highlights its potential to accelerate early recovery, aligning with its established use for over 15 years in pain and inflammation management. The consistent improvement in QoL scores across all postoperative days further underscores KT’s holistic benefits. However, the lack of a significant impact on trismus suggests that KT may not fully address the mechanical limitations post surgery. Future research should explore larger, blinded cohorts and longer follow-up periods to confirm these benefits and address limitations, such as potential bias and sample size constraints.
